# Expression of Staphylococcal Enterotoxins under Stress Encountered during Food Production and Preservation

**DOI:** 10.3390/toxins9120401

**Published:** 2017-12-15

**Authors:** Jenny Schelin, Yusak Budi Susilo, Sophia Johler

**Affiliations:** 1 Applied Microbiology, Department of Chemistry, Lund University, SE-221 00 Lund, Sweden; jenny.schelin@tmb.lth.se (J.S.); yusak.budi_susilo@tmb.lth.se (Y.B.S.); 2 Institute for Food Safety and Hygiene, Vetsuisse Faculty University of Zurich, Winterthurerstrasse 272, 8057 Zurich, Switzerland

**Keywords:** staphylococcal food poisoning, enterotoxin, *Staphylococcus aureus*, strain specific variation, regulatory circuits

## Abstract

Staphylococcal food poisoning (SFP) is the most prevalent cause of food-borne intoxications worldwide. Consumption of enterotoxins preformed in food causes violent vomiting and can be fatal in children and the elderly. While being repressed by competing bacteria in most matrices, *Staphylococcus aureus* benefits from crucial competitive advantages in foods with high osmolarity or low pH. During recent years, the long-standing belief in the feasibility of assessing SFP risk based on colony-forming units of *S. aureus* present in food products has been disproven. Instead, researchers and food business operators are acutely aware of the imminent threat arising from unforeseeable enterotoxin production under stress conditions. This paradigm shift led to a variety of new publications enabling an improved understanding of enterotoxin expression under stress conditions encountered in food. The wealth of data provided by these studies is extremely diverse, as it is based on different methodological approaches, staphylococcal strains, stressors, and enterotoxins. Therefore, in this review, we aggregated and critically evaluated the complex findings of these studies, to provide readers with a current overview of the state of research in the field.

## 1. Introduction

Staphylococcal enterotoxins (SEs) preformed by *S. aureus* during growth in food are a common cause of staphylococcal food poisoning (SFP). The European Food Safety Authority registered 434 reported SFP outbreaks in 2015, which equals more than half of all food-borne outbreaks associated with bacterial toxins [1]. In the US, the Centers for Disease Control estimate that 240,000 cases of SFP occur each year, leading to 1000 hospitalizations and six fatalities [2]. Shortly after oral intake of SEs, patients may suffer from nausea and violent vomiting, which is often accompanied by watery diarrhea, prostration, and moderate fever. Symptoms usually subside within 24 h, but may be fatal in children and the elderly with a fatality rate of 4.4% [3]. 

As the heat-stable SEs are preformed during growth of *S. aureus* in food and cannot be reliably inactivated through food processing [4], prevention of *S. aureus* growth and toxin formation in food is key to minimizing the risk of intoxication. However, assessing the SFP risk associated with a food product solely based on colony-forming units of *S. aureus* present has recently been shown to be unreliable [5,6]. While growth of the organism is repressed by competing bacteria in many food matrices, *S. aureus* exhibits a competitive growth advantage in foods with high sugar or salt concentrations or low pH. Understanding the effect of these stressors on enterotoxin expression is crucial to improving risk assessment and to adapting food production parameters to minimize the intoxication risk for the consumer. Within the last few years, a wide variety of different studies have tried to extend the very limited data available on the effect of food-related stressors on SE expression. In this review, we aim to subsume and critically discuss this wealth of novel findings in order to provide readers with a concise and current overview presenting the most striking insights.

## 2. Methodological Challenges in Quantifying SE Expression under Stress Conditions

Various approaches have been used to quantify SE expression under food-related stress conditions either on a transcriptional or translational level. In the past, the formation of SEs under stress conditions has mainly been assessed using immunological methods [7,8,9,10]. However, loss of serological recognition does not necessarily equal loss of emetic/biological activity [11]. 

Lately, there have been advancements in the development of non-immunologically based methods that provide promising ways of detection and quantification that will circumvent the dependency on serological recognition. Methods such as high-performance liquid chromatography (HPLC), reverse phase liquid chromatography coupled with ESI mass spectrometry (LC-ESI/MS), ultra-high-performance liquid chromatography-tandem mass spectrometry (UPLC-MS/MS) and liquid chromatography-mass spectrometry based on multiple reaction monitoring (LC-MS/MS (MRM)) have been developed and evaluated for detection of enterotoxins in different food matrices [12,13,14,15]. Although further optimization in the areas of, e.g., sample preparation and detection limits is still needed, these methods are a highly interesting and a promising complementation to existing techniques. By basing measurements on the intrinsic properties of the enterotoxin protein, these tools are independent of sometimes-unreliable antigen-antibody interactions. Thus, many problems associated with conventional immunological SE detection can be solved, including false positive/false negative reactions due to cross-reaction between serotypes, serological modifications of the enterotoxin, or simply breakdown of the antibodies.

Quantitative real-time PCR techniques determining enterotoxin mRNA levels open up a new range of possibilities to study enterotoxin gene expression under stress, and have been suggested to represent a useful tool in assessing the risk of SFP [16]. However, normalization using a sufficient number of reference genes validated under the specific stress conditions applied is of crucial importance. It is necessary to correct for variation introduced by differences in mRNA concentrations among samples, yield of RNA extraction/reverse transcription, as well as amplification efficiency [17]. Validated reference genes for normalization of qPCR assays involving NaCl, lactic acid, glucose and nitrite stress levels frequently encountered in food have been published [18,19,20].

Recent findings underline the necessity of confirming SE expression data generated using planktonic pure cultures by data obtained through measuring SE formation directly in the food matrix [5,21]. However, high-quality antibodies are not readily available for all SEs, and commercial detection kits are currently limited to the classical enterotoxins SEA-SEE, omitting the newly described SEs that are increasingly being associated with SFP outbreaks [22,23,24]. Immunological detection approaches in the food matrix, and particularly in cheese, may further suffer from low specificity and sensitivity, and false-positive test results have been reported due to matrix components or unspecific IgG binding, necessitating purification and concentration by affinity chromatography and dialysis [25].

Other important factors to consider when estimating or monitoring enterotoxin production in food is the actual virulence capacity of different *S. aureus* strains and whether there is a correlation between growth and toxin production in complex environments. Currently, the microbial safety guidelines of food products are determined by the acceptable concentration of viable cells (CFU per g or mL) present in a defined amount of food sample [26,27]. This is partly based on earlier studies demonstrating that a critical population size of 10^5^ CFU/g or mL is required for the presence of detectable amounts of enterotoxins [28]. Previous and later work have, however, demonstrated that prediction of enterotoxin concentrations produced in situ based on the number of viable cells is an unreliable indicator for predicting enterotoxin levels [6,20,23,29,30]. The study by Zeaki et al. [6] showed that even though all three natural isolates used in the study exhibited very similar growth patterns and rates over 14 days of incubation on pork sausages with or without lactic acid treatment, the levels of SEA produced, as well as the enterotoxin production rates, differed significantly. No apparent linkage between absolute cell number or growth rate and enterotoxin production/levels was detected, suggesting that assessment of the intoxication risk associated with a food item based solely on the level of *S. aureus* CFU detected is unreliable. A study on SFP outbreaks in the UK between 1969 and 1990 found different amounts of viable *S. aureus* cells, varying from not detectable to 1.5 × 10^10^ CFU/g, in the investigated food samples [31], a finding consistent with outbreak investigations elsewhere [23]. Combined, the aforementioned challenges emphasize the need for additional, more sensitive and robust tools for SE detection and quantification.

## 3. Effect of Food-Related Stressors on the Expression of SEA-SED

### 3.1. SEA

The effect of stress on *sea* prophage induction and SEA expression may vary, dependent on the pH, as well as the allelic *sea* variant and prophage present in the *S. aureus* strain tested [32,33]. Mild NaCl stress (2%) was reported to lead to phage induction and increased levels of *sea* gene produced in the food isolate Sa17, while at the same time resulting in lower SEA levels compared to BHI control conditions [34]. Mild acetic acid stress (pH 7–5.5) led to prophage induction and increased *sea* expression in Mu50 and food isolate Sa45, while no or very low levels of *sea* mRNA and SEA were detected at pH 5.0–4.5 [32]. Another study observed no effect of sorbic acid stress (0.15%, pH 5) on phage induction, as well as decreased SEA levels using *S. aureus* Sa17 [34]. Undissociated lactic acid (1.6 mM compared to 0.2 mM) was reported to be able to increase SEA production of strain cocktails grown in BHI broth [35]. However, treatment of pork sausage with 1–2% lactic acid was shown to be able to reduce SEA formation [6]. The effect of temperature on SEA production was reported to be strain dependent [36,37]. SEA production was detected at a temperature range of 10 °C to 45 °C [36,37], with SEA production rates increasing in most strains with increasing temperatures [37].

Co-culturing of *Lactococcus lactis* and *S. aureus* strain MW2 resulted in a slight improvement in maintaining *sea* expression in the stationary growth phase, whereas expression of other enterotoxin genes was impaired (*sec*, *sel*) or unaffected (*sek*, *seg*, *seh*) [38].

Several studies also provide data on SEA expression in meat products. Wallin-Carlquist et al. [5] determined SEA expression in boiled and smoked ham, Serrano ham, and black pepper salami over seven days. The meat product slices were each inoculated with 10^7^ CFU of *S. aureus* outbreak strain SA45. SEA levels detected in boiled and smoked ham after 1 day and in Serrano ham after 7 days of incubation were sufficient to cause SFP, with higher *sea* mRNA and SEA protein levels observed in boiled ham in comparison to smoked ham. No *sea* mRNA or SEA was detected in salami [5]. 

### 3.2. SEB

SEB expression is highest during the transition from exponential to stationary growth phase [39,40,41], coinciding with a peak in the activity of the quorum sensing system Agr (accessory gene regulator) [42,43]. It has been reported that SEB production is not affected by nitrite levels of up to 200 mg/L [8].

A recent study by Sihto et al. investigated the effect of NaCl (4.5%), nitrite (150 mg/L), glucose (30%), and lactic acid (pH 6.0) stress on *seb* promoter activity for transcriptional fusions of different *seb* upstream regions of clinical isolates in a USA300 and HG003 strain background [44]. The results of this study showed that mild stress conditions mimicking those encountered during food production and preservation can lead to significant changes in *seb* promoter activity, with glucose and NaCl stress reducing *seb* promoter activity, while lactic acid increased *seb* promoter activity. Interestingly, a significant effect of the strain background on *seb* promoter activation was shown, which by far surpassed the effect of different stressors on *seb* promoter activation. The activity of the same *seb* promoter in HG003 was far lower compared to USA300. However, deletion of *sigB* in HG003 led to an extreme increase in the activity of the *seb* promoter, suggesting that repression of the *seb* promoter in this strain is due to SigB activity [44].

### 3.3. SEC

There is only very limited data on the effect of food-related stressors on *sec* expression and, in particular, no data taking into consideration the different SEC variants. Even et al. demonstrated that *sec* expression is affected in mixed cultures with *Lactococcus lactis* held at a constant pH of 6.6 [38]. These conditions had almost no effect on *S. aureus* growth, but strongly affected expression of *sec,* as well as regulators *agr* and *sarA* in MW2*.* The expression of *sec* was also reported to be affected by glucose and NaCl stress [45,46]. Levels of *sec* mRNA and SEC protein were shown to be reduced 16-fold in conditions of 1.2 M NaCl compared to 0 M NaCl, independently of an intact *agr* [46].

### 3.4. SED

Recently, *sed* expression was studied in clinical isolates (RKI1, RKI2, SAI48, BW10, KLT8) under various stress conditions encountered during food production and preservation, including NaCl, sodium nitrite, lactic acid, and glucose stress (30%). Studies by Sihto et al. showed that NaCl (4.5%) and glucose stress can decrease *sed* expression, while lactic acid stress had no significant effect on *sed* expression [18,47]. Sihto et al. also reported that nitrite stress (150 mg/L) can lead to significantly induced *sed* mRNA levels, but does not result in higher SED protein levels [20]. First studies also conducted experiments quantifying *sed* transcription and SED protein formation in the food matrix [30,48]. Márta et al. [30] showed that *sed* is expressed in various ham products over an extended amount of time, with an increase in *sed* expression after five days. Susilo et al. [48] measured *sed* transcription and SED formation on boiled ham for 14 days at 22 °C in a wild-type strain (RKI1) and isogenic *agr* mutant, showing that loss of Agr only partially affects SED formation, even in a real food environment [48].

Interestingly, several studies measuring *sed* expression on both the transcriptional and translational level found that relative *sed* expression levels did not mirror the SED protein levels detected [20,48]. This may be due to regulation at the translational level. Alternatively, it could also reflect accumulation of solutes such as proline and glycine betaine under conditions of osmotic stress in order to maintain hydrostatic pressure, which has been hypothesized to result in impaired secretion of exotoxins [49]. Across studies, authors observed strain-specific variation in response to the different stressors and in the control of *sed* expression by regulatory elements. 

## 4. Regulation of SE Expression and Possible Over-Estimation of the Influence of Agr

The expression of SEA to SEE is regulated by a range of different mechanisms that to a varying extent are also interlinked with each other. Overall, two different groups can be distinguished: the prophage-encoded enterotoxins (SEA and SEE) and the *agr*-regulated enterotoxins (SEB, SEC, and SED). SEA expression and formation have been demonstrated to be quorum-sensing-independent, and regulation is instead linked to the life cycle of different *sea*-bearing prophages [33,34,50]. SEE is, in contrast, located on a defective prophage, and no impact of bacterial growth on *see* expression was found [41,51]. For SEB, SEC, and SED, it has been well established that expression is directly or indirectly positively regulated by the global regulator *agr*, as well as other global regulatory systems [41,45,52,53,54,55]. Agr is a well-characterized two-component quorum-sensing system in *S. aureus* [54,56,57,58]. The *agr* operon encodes three transcripts, RNAI, RNAII, and RNAIII [59]. RNAII encodes for proteins that constitute the quorum-sensing part of the system (AgrA, B, C, and D). This provides signals for the transcription of RNAIII, depending on the density of the bacterial population. RNAIII functions as a regulatory signal and activity indicator of the Agr system, and contains the δ-hemolysin (*hld*) gene [59,60]. Both RNAII and RNAII I transcripts are weakly transcribed in the early exponential phase of growth followed by increased expression in the early stationary phase [59,61,62]. Consequently, when RNAIII levels are low, *S. aureus* cells produce more cell-wall associated proteins such as protein A, coagulase and fibronectin-binding protein at the beginning of growth in order to establish attachment to the substrate or host cells. Later, in the early stationary phase, the RNAIII transcript level increases in response to higher cell densities. This leads to a decrease in the transcription of cell-surface proteins and an increase in the transcription of exotoxin proteins such as *α*-toxin, *β*- and *δ*-hemolysin, TSST-1, and enterotoxins [43,57,59,60,63]. For SEB and SED, the Agr-mediated effect is indirect, with RNAIII increasing the transcription by repression of the repressor of toxins (Rot), an RNAIII-binding protein that is a member of the *S. aureus* Sar-family of transcriptional factors [64]. The effect of *agr* on SED has been rather well studied, and even though an indirect impact on expression levels has been described, several studies have also shown that the role of *agr* might have been overestimated [53,65].

It has been reported that loss of the Agr system causes 82% (5-fold) reduction in SED formation in an in vitro study comparing *agr^−^* and *agr^+^* derivatives (ISP546 and RN4220) of the SigB-deficient *S. aureus* NCTC8325 strain [53]. Tseng and co-workers later identified specific *sed* promoter elements important in Agr control [65]. They reported a 1.7-fold decrease of *sed* promoter activity in an Agr deficient mutant (Agr^−^ Rot^+^) compared to its isogenic parental strain (Agr+ Rot+), and were able to show that regulation of *sed* gene transcription was mediated through control of Rot activity by Agr. However, already at this stage, the importance of the Agr system on enterotoxin expression is suspected to be overestimated due to the use of the laboratory strain *S. aureus* NCTC8325. This strain has a defect in the *sigB* operon that affects Agr, Rot, and Sar global regulators, and has been reported to increase the expression of RNAIII [66,67,68,69]. The effect of loss of Agr on SED expression was recently further studied using clinical *S. aureus* isolates [20,47], and growth in both defined laboratory medium and a food matrix. Growth in Luria Bertani (LB) broth with or without salt stress revealed no significant differences in SED concentration in three *S. aureus agr* deletion mutants compared to isogenic wild types. Interestingly, for SEB, loss of *agr* was found to significantly decrease promoter activity in another study performed in LB, using strains with intact *sigB* operon, underlining the importance of considering both strain- and enterotoxin-specific variation in *S. aureus* [44].

Studies investigating the influence of *agr* on SE expression have so far almost exclusively relied on planktonic growth in cultivation broth, which only partially reflects conditions encountered in the food matrix. A recent study investigated, for the first time, the effect of *agr* on *sed* transcript levels in a *S. aureus* food isolate directly in the food matrix [48]. The study showed that *sed* transcript levels of an *agr* mutant were similar to its isogenic wild type when grown on boiled ham. This strongly supports previous findings suggesting that regulation of SED is not as strongly dependent on the Agr system as previously believed, and that high concentrations of SED may be produced independently of *agr* [54].

## 5. Conclusions

Taken together, the above compiled findings regarding the effect of different food-related stressors underline the importance of further studies directly in the food matrix. Rather than laboratory strains, strains isolated from food samples and clinical cases of SFP should be used in order to account for the influence of a complex growth milieu combined with the pronounced strain-specific variation observed in *S. aureus*. Additional studies on the impact of regulatory elements will also be crucial in understanding the complex, intertwined regulatory networks controlling SE expression. Through an ongoing expansion of data concerning *S. aureus* growth behavior and SE production, we can improve risk assessment and consumer safety by supporting food business operators in the adaptation of food production processes and formulations in order to maximize food safety. Due to the complex effects of stressors on the production of various enterotoxins, these adaptations need to be tailored to and evaluated for each specific production process.
